# Diesel siphoner's lung: An unusual cause of hydrocarbon pneumonitis

**DOI:** 10.1002/ccr3.3545

**Published:** 2020-11-18

**Authors:** Tirtha Man Shrestha, Suraj Bhatta, Ramesh Balayar, Sagar Pokhrel, Pankaj Pant, Gaurav Nepal

**Affiliations:** ^1^ Department of General Practice and Emergency Medicine Tribhuvan University Teaching Hospital Maharajgunj Nepal; ^2^ Maharajgunj Medical Campus Tribhuvan University Institute of Medicine Maharajgunj Nepal; ^3^ Department of Pulmonology and Critical care Tribhuvan University Teaching Hospital Maharajgunj Nepal

**Keywords:** aspiration, diesel, hydrocarbon, pneumonitis

## Abstract

The practice of manual siphoning of diesel from fuel tanks is common among automobile mechanics in Nepal. When an automobile mechanic with a history of diesel siphonage presents with respiratory symptoms, the diesel siphoner's lung diagnosis should be considered. Clinical suspicion confirmed by radiological findings can help in early management and prevention of permanent damage.

## Graphical abstract

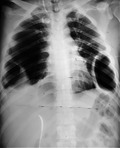

## 1 INTRODUCTION

Hydrocarbon pneumonitis following unintentional aspiration of volatile compounds such as diesel is an uncommon and under‐reported emergency medical condition. Most cases of hydrocarbon pneumonitis are associated with accidental aspiration in children and occupational exposure in fire‐eaters. However, in South Asian countries, diesel siphoning from fuel tanks is a common practice for automobile mechanics/garage workers, especially in rural communities. Fuel siphoning involves using a nozzle and a simple rubber tube to siphon diesel fuel from a car tank or fuel drum.^1^ This siphoning method may result in accidental intake and inhalation of diesel fuel. Although ingestion usually causes transient vomiting, diarrhea, and abdominal pain, diesel fuel's inhalation destroys surfactants, reduces lung compliance, and causes direct inflammation in the lungs. The inhaled diesel quickly reaches the alveoli without causing any apparent coughing but triggers a robust inflammatory response in the lung parenchyma.^2^, ^3^ Without a proper history of exposure, such patients may be misdiagnosed as tuberculosis or bacterial pneumonia, resulting in faulty treatment and poor outcomes. Therefore, we herein report the case of a young adult who developed chemical pneumonitis with bilateral pleural effusion and pneumothorax following diesel siphoning. Since siphoning is a common practice in Nepal, this report will help increase the value of occupational history taking and make clinicians more vigilant when people working in the garage present with respiratory symptoms.

## 2 CASE REPORT

A 24‐year‐old man, a newly appointed automobile mechanic, presented to the emergency room of Tribhuvan University Teaching Hospital with a chief complaint of progressive shortness of breath, low‐grade fever, bilateral chest pain, and nonproductive cough for three days. The shortness of breath was exertional, and the patient did not complain of any orthopnea, paroxysmal nocturnal dyspnea, or pedal edema. The low‐grade fever occurred three days before dyspnea, had a Tmax of 99.8°F, was on and off, and was not associated with chills or rigors. There was no history of wheezing, sputum production, hemoptysis, night sweats, weight loss, or anorexia. He denies any rash, joint pain, numbness, or weakness in the extremities. On detailed inquiry, he revealed that he recently started working as an automobile mechanic and had to aspirate diesel from fuel tanks often. As he was new to this job, he had accidentally aspirated a small volume of diesel several times in the last two weeks. He was a nonsmoker and had never consumed alcohol in his lifetime. The patient had no past medical history of note, no history of allergies, and had not taken any drugs or medications recently.

On examination, he was ill‐looking with a temperature of 98°F, a sphygmomanometric blood pressure of 140/90mmHg, a heart rate of 110 beats/minute, and a respiratory rate of 28/minute saturating poorly in ambient air (peripheral SpO_2_ 87%, as measured by pulse oximetry). He had no pallor, icterus, lymphadenopathy, cyanosis, clubbing, edema, and dehydration. There was a decreased excursion of the chest wall on chest examination, diminished breath sounds bilaterally along with diffuse bilateral basal crepitations, and a dull stony percussion note bilaterally. The examination of other systems did not reveal any abnormalities. The patient had leukocytosis with a total WBC count of 13900/mm^3^, and differential leukocyte count consisted of 90% neutrophils and 10% lymphocytes. Hemoglobin, hematocrit, and platelet count were normal. Renal function test, liver function test, coagulation assay, and serology tests were regular. Arterial blood gas analysis was within the reference range.

**Figure 1:**
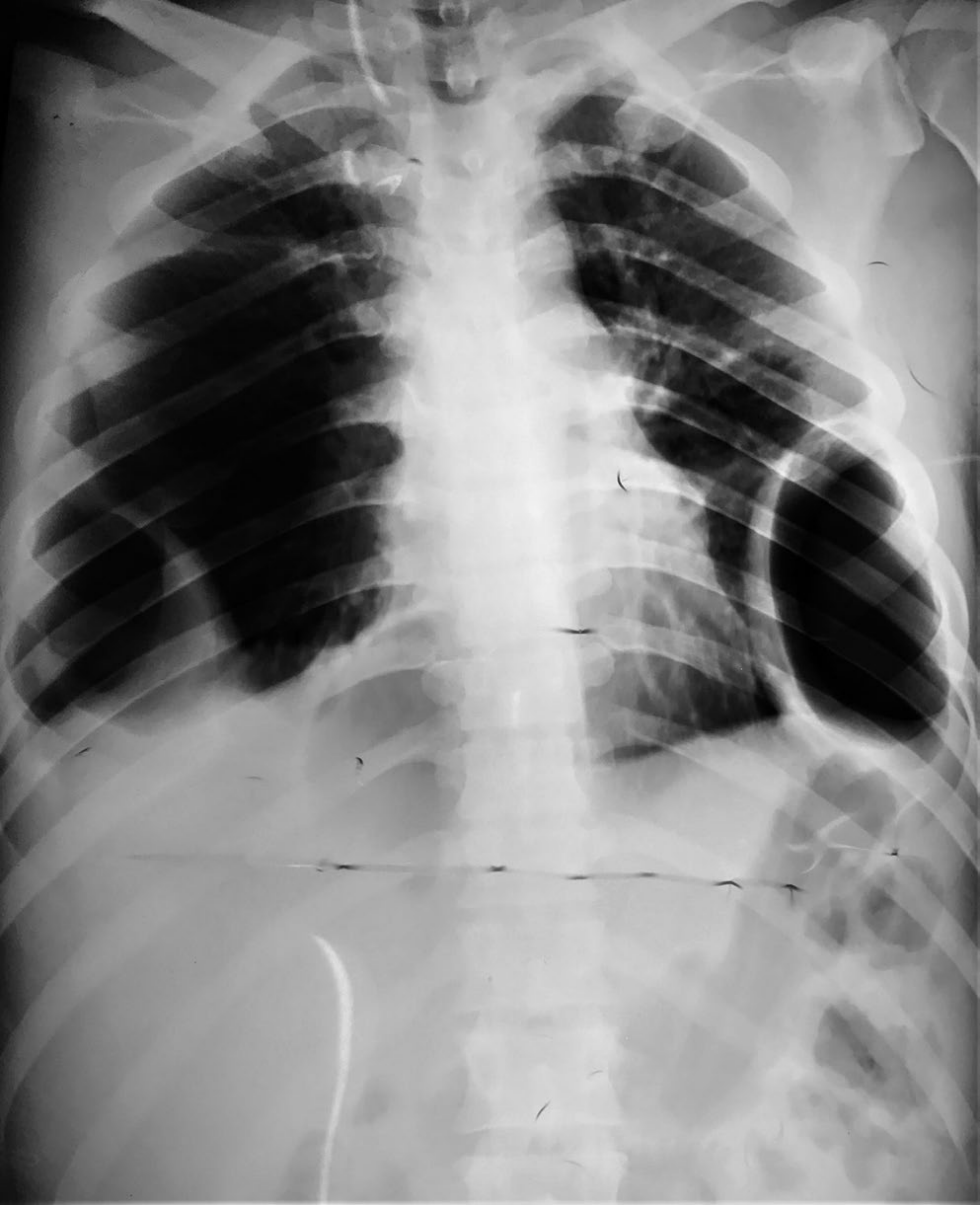
Chest X‐ray supine AP view showing pneumonic consolidations over bilateral lung field with bilateral pleural effusion and pneumothorax

**Figure 2:**
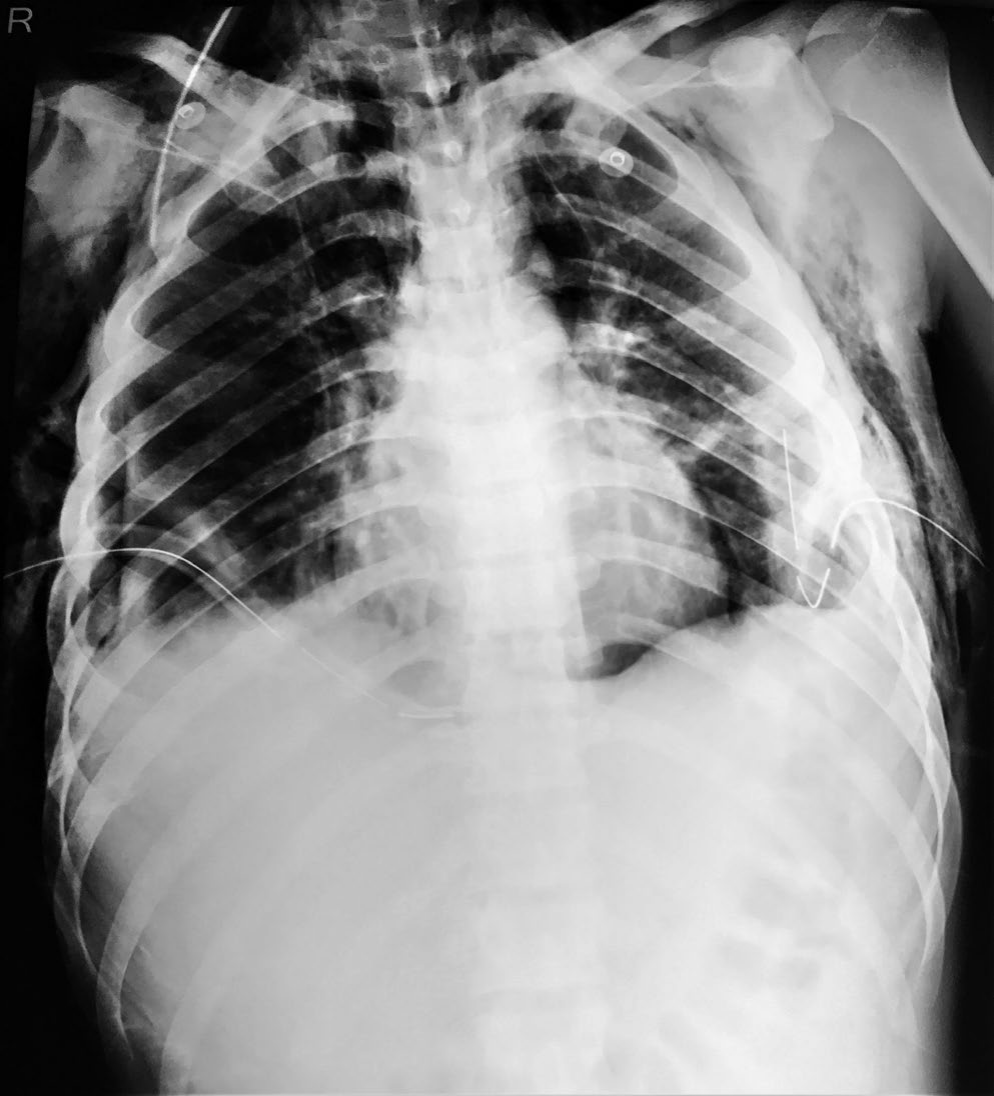
Chest X‐ray supine AP view showing pneumonic consolidations over bilateral lung field with bilateral pleural effusion, right‐sided pneumothorax, and bilateral surgical emphysema

Chest X‐ray (CXR) done at admission (Figure 1) showed pneumonic consolidation over the bilateral lung field with bilateral pleural effusion and pneumothorax. Chest tubes were inserted bilaterally under ultrasound guidance to alleviate pleural effusion and pneumothorax. On the second day of chest tube insertion, CXR (Figure 2) was done again, revealing the left lung's expansion, persistent right‐sided pneumothorax, and bilateral surgical emphysema. Contrast‐enhanced computed tomography of thorax revealed right‐sided pneumothorax, bilateral pleural effusion, bilateral patchy consolidated lung parenchyma, and bilateral chest tube in situ.

**Figure 3:**
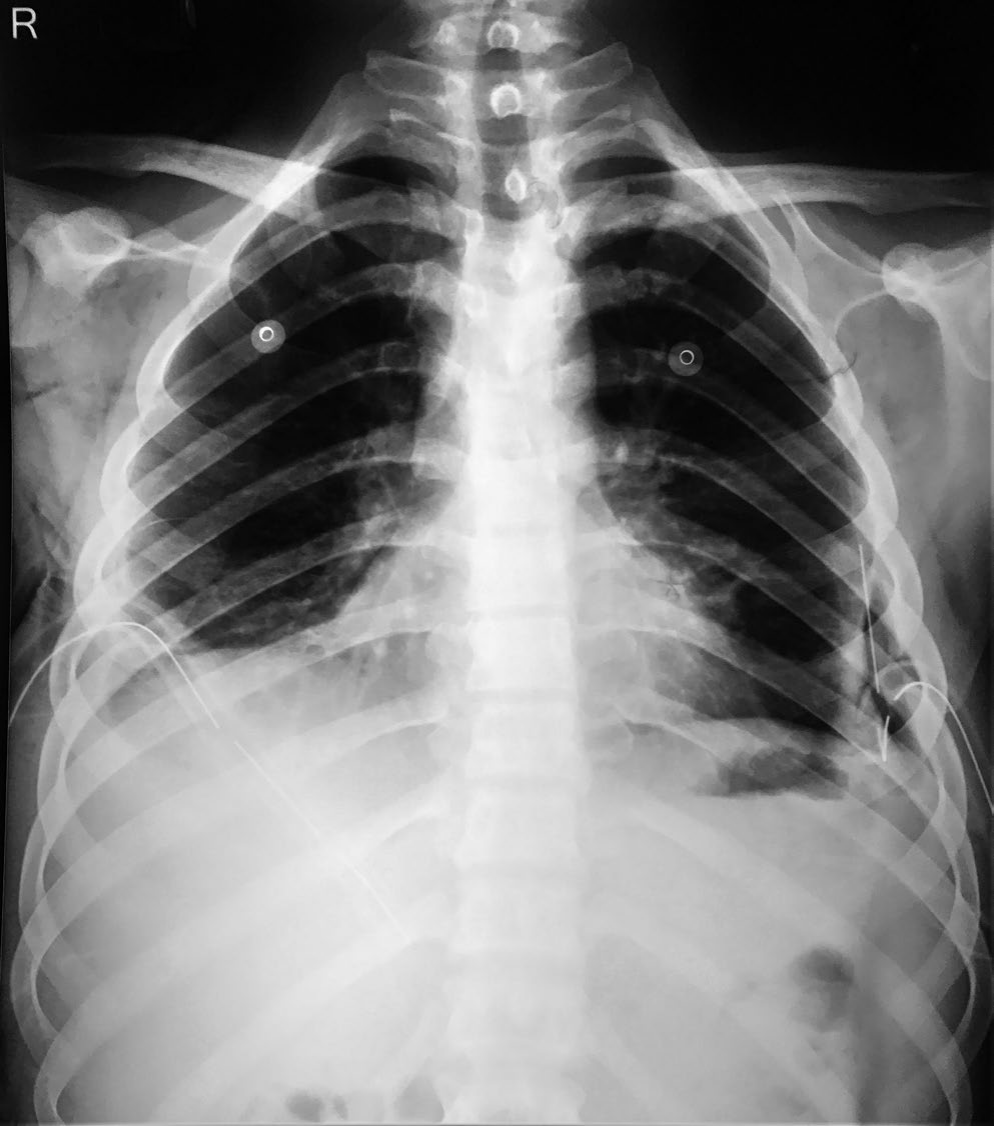
Chest X‐ray erect PA view showing resolution of patchy peripheral pneumonitic changes, resolution of pneumothorax, and surgical emphysema resolution. However, bilateral pleural effusion is still present

Owing to radiological findings, clinical features, and siphonage history, the diagnosis of hydrocarbon pneumonitis was made. Since Nepal is a tuberculosis endemic region, pulmonary tuberculous was also considered as alternative diagnosis. However, sputum examination did not reveal the acid‐fast organism; neither did the GeneXpert PCR test. The patient was kept under intravenous piperacillin/tazobactam, nebulization was done six hourly, and high flow oxygen was administered. The patient developed no complications during his hospital stay. The patient was discharged after two weeks when there was significant radiological regression of consolidations (Figure 3), improvements in pulmonary symptoms, and maintenance of oxygen saturation above 90% in room air. The patient was advised to follow‐up regularly and is doing well at the time of writing.

## 3 DISCUSSION

Lipoid pneumonia is a rare form of lung disease first defined in 1925 by Laughlin and is due to fat‐containing products accruing in the distal airways and alveoli, leading to an inflammatory reaction that hinders gas exchange. Based on the source of lipids, it can be categorized as exogenous and endogenous lipoid pneumonia.^4^, ^5^ Hydrocarbon pneumonitis following aspiration of diesel is a form of exogenous lipoid wherein aspirated diesel reaches the alveoli rapidly without evoking any significant cough but initiates an intense inflammatory reaction in the pulmonary parenchyma. This is a rarely described clinical scenario, although diesel siphonage from automobiles and fuel drums is a common practice in developing countries such as Nepal.^6^ Other hydrocarbon compounds with low viscosity and high volatility, such as kerosene, turpentine, gasoline, naphthene, or aromatic classes, can also cause hydrocarbon pneumonitis when inhaled accidentally or intentionally.^7^ Children, adolescents, or young adults, and workers with constant exposure to fuels are particularly at risk.^8^

Aspirated oil particles are generally bland, nonirritating, and do not stimulate the cough reflex pathway and reach the lower airways. Once in the airways, they impair mucociliary clearance, and this further impairs their expulsion. Once the hydrocarbon product is aspirated, it is phagocytized by interstitial‐alveolar macrophages, causing their activation and initiating an alveolar interstitial inflammatory response, which produces an acute chemical pneumonitis.^9^ The emulsified and engulfed hydrocarbon by alveolar macrophages can remain inside for a long time, which are released into the alveoli in a timely fashion following disruption of the macrophages, inciting a giant cell response leading to fibrosis and disruption of bronchial and alveolar structure.^10^ Rarely, cavitation may be observed with parenchymal destruction around large lipid‐containing vacuoles. It is estimated that even < 1ml of hydrocarbon is sufficient to induce lung injury.^11^

The acute presentation can simulate infectious pneumonia with fever, cough, and pleuritic chest pain within a few hours following accidental aspiration of kerosene or diesel while siphoning, but can also mimic acute respiratory distress syndrome usually following a massive exposure.^6^,^12^, ^13^ As in our case, chronic presentations include insidious onset shortness of breath, intermittent fever, persistent cough, weight loss simulating chronic infections, or interstitial lung disease. Other features may include cyanosis, clubbing, recurrent infections, hyperthermia, and unresolving radiological features of pneumonia.^12^

Diagnosis of patients who may have undertaken fuel siphonage depends on three criteria: the presence of pulmonary symptoms following an episode of fuel siphonage, typical manifestations on radiological investigations with suspected history, and lipid‐laden macrophages on BAL or pathologic findings.^2^ The definitive diagnosis is made by demonstrating lipid‐laden macrophages in BAL fluid and the alveoli or interstitium in bronchoscopic lung biopsy.^6^ However, the unfamiliarity with this condition, the absence of specific clinical‐radiological features makes diagnosis difficult unless a strong suspicion is held and a thorough history is obtained.^6^ Nevertheless, the recognition of this entity is essential for several reasons. In cases with suspected infection, unnecessary antibiotic treatment can be obviated unless there is suspicion of superimposed infection. In instances of a mass lesion, invasive procedures can be avoided. Also, recognizing it can prompt additional history to identify the culprit and prevent associated complications such as pulmonary fibrosis, superimposed infection, and cor pulmonale.^14^ Also, in TB endemic region like ours, young patients with a history of a cough, shortness of breath, and pneumothorax on chest X‐ray are empirically given antitubercular therapy, without any clinical features or investigations suggestive of the same, which warrants a detailed history taking and performing necessary investigations.^15^

Hydrocarbon pneumonitis treatment is fundamentally symptomatic, even in cases with significant lung involvement.^16^ These include respiratory support and prevention of complications, which are sufficient in most cases. Corticosteroids and antibiotics should not be used routinely as a prophylactic measure as there is no good supporting evidence for the use of either.^8^ The prognosis of hydrocarbon pneumonitis is generally good since lung lesions are usually reversible with lung involvement resolution in weeks or months.^17^

## 4 CONCLUSION

The practice of manual siphoning of diesel from fuel tanks is common among automobile mechanics in Nepal. The diagnosis of diesel siphoner's lung or hydrocarbon pneumonitis should be considered when a mechanic or garage worker with a diesel siphonage history presents with compatible respiratory symptoms. A high index of clinical suspicion with confirmation by radiological findings can help in early management and permanent damage prevention. Along with clinicians, awareness of this condition is also equally crucial for mechanics. Community intervention to raise awareness regarding this disease among mechanics is of utmost importance, and the practice of mineral oil siphonage should be abandoned permanently.

## 5 ETHICS APPROVAL AND CONSENT TO PARTICIPATE

There is no need for ethical approval for a case report according to the local ethical guidelines. Written informed consent was taken from the patient in the Nepali language to include his clinical details in this article.

## CONFLICT OF INTEREST

None declared.

## AUTHOR CONTRIBUTIONS

TMS, GN, and PP: established the diagnosis. SP, SB, and RB: reviewed the literature and designed the manuscript. GN and TMS: redrafted the manuscript in its current form. All authors read and approved the final version of the manuscript.

## Data Availability

No dataset was generated in this study.
